# A Case for Specific Education of Advanced Practice Providers in Allergy & Immunology: Results of a Gap Analysis and Targeted Needs Assessment

**DOI:** 10.12688/mep.20586.1

**Published:** 2024-10-17

**Authors:** Maureen Bauer, Chad Stickrath, Dan Atkins

**Affiliations:** 1 Department of Pediatric Allergy & Immunology, Children’s Hospital Colorado, University of Colorado School of Medicine, Aurora, Colorado, USA; 2 Department of Internal Medicine, University of Colorado School of Medicine, Aurora, Colorado, USA

**Keywords:** Advanced Practice Provider Education, Nurse Practitioner, Physician Assistant, Allergy & Immunology

## Abstract

**Introduction:**

Advanced practice providers (APPs) are increasingly utilized throughout the health care system. At present there is no nationally sanctioned curriculum for APPs providing Allergy & Immunology (A&I) care.

**Methods:**

A nationwide gap analysis and targeted needs assessment was conducted to examine the current level of education/training of APPs in A&I.

**Results:**

At present, training in A&I in NP/PA school is quite limited. The formal education and clinical training APPs receive at practice sites varies significantly with most APPs feeling only somewhat comfortable providing A&I care upon completion of their training period.

**Conclusions:**

Results of a nationwide gap analysis and targeted needs assessment identify the need for providing education specific for A&I to APPs practicing in this field.

## Practice points

- At present there is no national curriculum specific to Advanced Practice Providers (APP) in Allergy & Immunology.
- APPs report a lack of training specific to A&I in Nurse Practitioner/Physician Assistant School.
- There is a significant range in the quantity and quality of formal education and clinical training of APPs regarding A&I.
- The majority of APPs reported being only somewhat comfortable in providing A&I care after completion of their training and cited a need for a dedicated curriculum.

## Introduction

Advanced Practice Providers (APPs), (Nurse Practitioners (NP), and Physician Assistants (PA)) are increasingly utilized throughout the health care system ^1^. This is likely due to a combination of factors including physician shortages, the shorter training time required to become an APP, the lack of a residency requirement and the increasing number of organizations offering NP/PA programs ^1^. While the largest increase is in primary care, the number of APPs entering specialty practices increased by 22% from 1008–2016 ^2^.

At present a nationally sanctioned A&I curriculum specific to APPs within A&I is lacking, likely due in part to state-by-state regulatory requirements that dictate the scope of APP practice ^3, 4^. A prior needs assessment of practicing APPs at Children’s Hospital Colorado demonstrated that the majority of APPs felt only somewhat comfortable managing their current patients, highlighting a knowledge gap that may exist nationally as well ^5^.

## Methods

### Ethics

This educational project was determined to be exempted from IRB oversight by the University of Colorado School of Medicine. Given the study was deemed exempt, written informed consent was not required, as this was an educational project in which sensitive information was not obtained. 

To examine the current state of education/training for APPs within A&I on a national level, a gap analysis and targeted needs assessment were performed with recruitment of APPs on a national level through the American Academy of Allergy, Asthma & Immunology (AAAAI) Allied Health List serve. This was done as an initial step in development of a Core Curriculum for APPs within A&I through the AAAAI educator development award. The gap analysis consisted of a series of 20 qualitative interviews with APPs and 5 physicians (who had recently trained APPs in A&I) using open ended questions to better understand the current level of education/clinical training. A balance of APPs practicing in academic medical centers and private practices, in addition to years of experience in A&I was intentionally included in the gap analysis. A targeted needs assessment was generated via the modified Delphi method from topics identified by the gap analysis.

## Results

Results of the gap analysis identified that none of the APPs received significant education specific to A&I in NP/PA school. There was a wide range of education received at practice sites (1–2 weeks to 24+ months) and no APP reported receiving a structured curriculum specific for their role. Among physicians, most reported a strong need for an APP dedicated curriculum with physicians typically modifying lectures initially prepared for physician fellows to a level more appropriate for APPs, while noting this was suboptimal. The time needed to train an APP was also considered significant which was difficult to balance with ongoing clinical needs. Fifty-six APPs within the AAAAI Allied Health Committee completed the targeted needs assessment out of 140 total members (40% completion rate). Responding APPs’ experience in A&I ranged from <1 year (4%), 1–3 years (8%), 4–5 years (18%), 6–10 years (25%) to 11+ years (45%). Fifty four percent of APPs were practicing in academic medical centers, 35% in private practice and the remainder selected other. When assessed on a 5-point Likert Scale if training specific to A&I in NP/PA school prepared them for clinical practice the overwhelming majority strongly disagreed (35%) or somewhat disagreed (32%). Most APPs had no lectures specific to allergy (18%) or only 1–2 lectures (52%) in NP/PA school. Regarding immunology, the majority of APPs reported only 1–2 lectures (50%) in NP/PA school with 32% reporting none.

**Figure 1.**
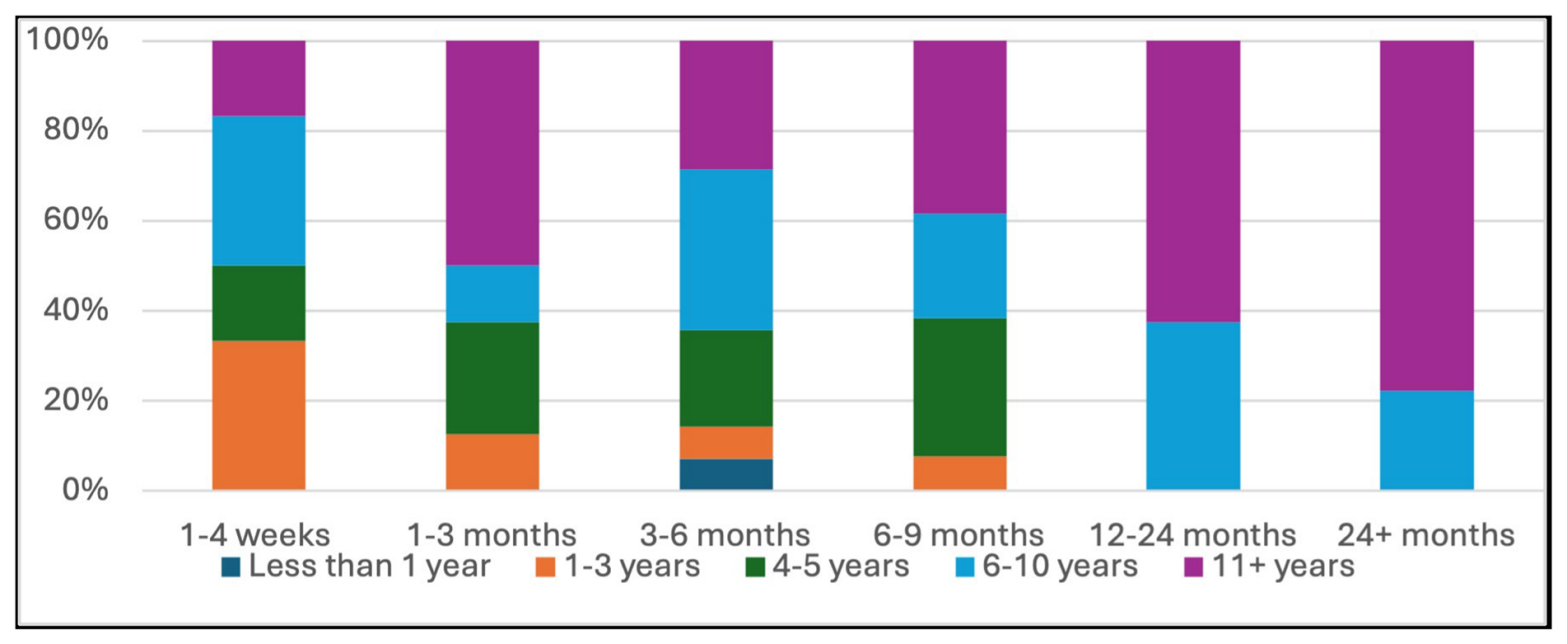
Bar graph comparing duration of clinical training to years in the field.

The amount of formal education (didactics, journal clubs etc.) received at practice sites when starting as an APP in A&I, ranged widely from none (18%), to limited (1–4 lectures, 24%), moderate (5–10 lectures, 16%), to extensive (10+ lectures, 42%). The duration of clinical training also varied significantly, with training periods of 0–4 weeks (14%), 1–3 months (12%), 3–6 months (26%), 6–9 months (20%), 12–24 months (12%) and 24+ months (16%). Interestingly, there was a trend towards less time in clinical training amongst APP’s who more recently entered the field of A&I (see Figure 1). When comfort level at completion of their training period was assessed via a 5-point Likert scale, the majority felt somewhat comfortable (52%), with 4% being extremely uncomfortable, 14% somewhat uncomfortable, 4% neither and 26% extremely comfortable. When asked qualitatively what was missing from their training experience in A&I, frequent responses included a dedicated curriculum for APPs, continuing education specific to APPs in the field and an APP fellowship.

## Discussion

Results of a gap analysis and targeted needs assessment identified that training in A&I in NP/PA school is quite minimal, consisting of 1–2 lectures only reported by most respondents. It is essential that hiring physicians/institutions are aware of this. However, depending on the state in which an APP may be licensed for independent practice, additional education/training in A&I would likely be needed. The range of formal education each APP received varied significantly, as did the duration of post-graduate clinical training, with most APP’s reporting they only felt somewhat comfortable after completion of training, which likely represents a significant training gap. 

Given the scope of practice of an APP varies based on state regulations and needs of the individual practice/institution, it’s difficult to determine the optimal duration/rigor of training. However, given the identified lack of significant training in A&I in NP/PA school, one would presume that optimal clinical training should be on the order of months/years as opposed to weeks. A frequently cited educational need in both the gap analysis and targeted needs assessment was for an APP fellowship specific to A&I. APP fellowships have been developed for other specialties including hematology/oncology, neonatology, surgical subspecialities, hospital medicine etc ^6– 8^. The duration of these programs varies but typically last 6–24 months ^8^. Therefore, a similar duration of training in A&I is likely needed.

Interestingly, there was a statistically significant trend towards APPs who more recently graduated NP/PA school having less clinical training before practicing at their current level of independence than those who have been in practice 6 or more years. While the reasons for this are unclear, financial and logistical factors such as patient wait times and physician bandwidth to train an APP may be contributing. As APPs are not receiving significant training in A&I in NP/PA school it is unlikely to be due to a stronger A&I knowledge base when entering the field.

There are limitations of this study. Participants were recruited from the AAAAI Allied Health Committee which may not represent all APPs in private practice. However, the gap analysis participants were intentionally balanced for site of practice and 35% of those who completed the targeted needs assessment were in private practice. Similarly, a large portion of the respondents were highly experienced in the field with 45% being in A&I for 11+ years which also likely impacts responses. 

In conclusion, results of a gap analysis and targeted needs assessment of APPs in A&I identify the lack of training specific to A&I in NP/PA school with a wide range in formal education and clinical training occurring across the country. The majority of APP’s only felt somewhat comfortable in their role after completion of training which supports the development of a curriculum specific to APPs within A&I on the national level.

## Ethics and consent

This educational project was determined to be exempted from IRB oversight by the University of Colorado School of Medicine. As for the IRB exemption, it was determined on 11/5/2024. Since they exempted it from research ethics review there is no IRB reference number but the internal number within the IRB for the request is #255054. Given the study was deemed exempt, written informed consent was not required, as this was an educational project in which sensitive information was not obtained. 

## Data Availability

Dryad: Needs assessment of advanced practice provider education in allergy & immunology, https://doi.org/10.5061/dryad.fxpnvx127 ^9^ This project contains the following underlying data: Data for Publication. Updated README Data are available under the terms of the Creative Commons Zero "No rights reserved" data waive (CC0 1.0 Public domain dedication).
