# A Case for Specific Education of Advanced Practice Providers in Allergy & Immunology: Results of a Gap Analysis and Targeted Needs Assessment

**DOI:** 10.12688/mep.20586.2

**Published:** 2025-04-03

**Authors:** Maureen Bauer, Chad Stickrath, Dan Atkins

**Affiliations:** 1 Department of Pediatric Allergy & Immunology, Children’s Hospital Colorado, University of Colorado School of Medicine, Aurora, Colorado, USA; 2 Department of Internal Medicine, University of Colorado School of Medicine, Aurora, Colorado, USA

**Keywords:** Advanced Practice Provider Education, Nurse Practitioner, Physician Assistant, Allergy & Immunology

## Abstract

**Introduction:**

Advanced practice providers (APPs) are increasingly utilized throughout the health care system including in subspeciality practices.

**Methods:**

A nationwide preliminary gap analysis and targeted needs assessment was conducted to examine the current onboarding/clinical training experiences of APPs in Allergy & Immunology (A&I).

**Results:**

At present, training in A&I in NP/PA school is typically limited. The onboarding/clinical training APPs receive at practice sites upon entering A&I varies with most APPs feeling only somewhat comfortable providing A&I care upon completion of their training period.

**Conclusions:**

Results of a nationwide gap analysis and targeted needs assessment identify the need for further education specific for APPs in A&I and consideration of standardization of the onboarding process at clinical practice sites.

## Disclosure

This project was funded through the American Academy of Allergy, Asthma & Immunology (AAAAI) Educator Development Award. The authors do not have any relevant disclosures. 

## Practice points

- At present there is no dedicated residency/fellowship for Advanced Practice Practitioners in Allergy & Immunology.
- There is a significant variation in the onboarding processes new APPs to A&I experience.
- The majority of APPs reported being only somewhat comfortable in providing A&I care after completion of their onboarding and cited a need for additional educational efforts specific to their role.

## Introduction

Advanced Practice Providers (APPs), (Nurse Practitioners (NP), and Physician Assistants (PA)) are increasingly utilized throughout the health care system ^1^. This is likely due to a combination of factors including physician shortages, the shorter training time required to become an APP, the lack of a residency requirement and the increasing number of organizations offering NP/PA programs ^1^. Literature demonstrates NPs and PAs provide high level quality and cost-effective care in various medical settings ^2– 5^. Of note, while PAs and NP’s may serve similar purposes in certain medical settings, there are differences in their training, certification and ability for independent practice ^6^. For example, at present, NPs can practice independently from a physician in 27 states while only 3 states allow full independent practice for a PA ^7, 8^. States with large rural populations tend to allow for more independent practice with this being associated with improvements in access to primary care and decreased emergency department use ^9^. While the largest increase of APPs is in primary care, the number of APPs entering specialty practices increased by 22% from 2008–2016 ^10^.

Like medical school for physicians which requires additional training after graduation, transitions to practice (TTP) programs for NPs and PAs are becoming increasingly common ^6, 11^. TTP programs typically consist of fellowship/residencies or onboarding programs with fellowships lasting 1–2 years and onboarding programs typically ranging typically between 6 months to 1 year ^6^. Allergy & Immunology (A&I) is a subspeciality in which there is currently no published formal residency or fellowship for APPs ^6^. The purpose of this project was to perform a preliminary assessment of current onboarding/educational experiences of APPs practicing in A&I to inform development of a national core curriculum specific to APPs.

## Methods

### Ethics

This educational project was determined to be exempted from IRB oversight by the University of Colorado School of Medicine. Given the study was deemed exempt, written informed consent was not required, as this was an educational project in which sensitive information was not obtained. The project was funded through the AAAAI (American Academy of Allergy, Asthma & Immunology) Educator Development Award.

To examine the current state of education/onboarding for APPs within A&I on a national level, a gap analysis and targeted needs assessment were performed with recruitment of APPs through the American Academy of Allergy, Asthma & Immunology (AAAAI) Allied Health List serve. Any APP within the AAAAI was eligible to participate. Given this project was supported through the AAAAI, the authors only had access to this group of Allied Health individuals, although this may not be a nationally representative sample. As there is no central accrediting body for APPs practicing specifically within A&I as there is for physicians (The American Board of Allergy & Immunology) the number of APPs practicing within A&I is not documented. There are other academic societies within A&I that also have Allied Health memberships which the authors did not have access to survey, which may limit the generalizability of results. 

Qualitative Assessment: A gap analysis was conducted which consisted of a series of 20 qualitative interviews with APPs practicing within A&I and 5 physicians who had recently onboarded APPs in A&I at their clinical practice site. As A&I is a small field, many practices may not have an existing experienced APP to assist with onboarding, requiring that physician onboard/train APPs new to A&I.

Qualitative interviews consisted of open ended questions to better understand the current experience of education/onboarding within A&I specifically. For example, participants were queried on their training specific to A&I during NP/PA school and their onboarding/educational experience upon entering A&I (Full script available in Supplement 1). Physicians were similarly asked about their experiences training/onboarding APPs who were new to A&I. Individuals were recruited from the AAAAI Allied Health List Serve, which as previously mentioned, may limit generalizability. We sought to include APPs practicing in both academic medical centers and private practices, in addition to the number of years of experience in A&I was intentionally included in participants in the gap analysis.

Quantitative Assessment: Following qualitative interviews in the gap analysis, a targeted needs assessment was generated via the modified Delphi method from topics identified in the gap analysis. This survey was sent to the AAAAI Allied Health List serve. Topics included the duration of time practicing within A&I, practice setting, quantitative information on training specific to A&I in NP/PA school, duration of clinical training during onboarding to A&I, formal education during onboarding, comfort level upon completion of onboarding and an open ended question on what was lacking from the onboarding process (Full survey available in Supplement 1). Of note, given an ultimate goal to develop a core curriculum that is applicable to all APPs on a national level regardless of state licensure/ability for independent practice, detailed information on practice by state was not captured nor was data captured based on NP or PA designation. Due to this being an IRB exempt study, indirect identifiers such as age, city or state of residence and gender were also not captured.

## Results

Qualitative Results: Twenty APPs completed qualitative interviews in the gap analysis. Participants had been practicing in A&I for a range of 1–20 years with 60% of participants in academics and 40% in private practice. APPs were in practices/institutions with a range of 1–20 other APPs in A&I although most respondents were in settings with 1–3 APPs in A&I total, reflective of the small size of the subspeciality. Regarding training specific to A&I in NP/PA school, the majority reported receiving 1–2 lectures (typically on asthma guidelines). 

There was a wide range of education/onboarding received at practice sites upon entering the field. Clinical experiences ranged from 1–2 weeks to 24 months prior to practicing at current level of independence. No APP reported receiving a structured curriculum specific for the APP role during onboarding. Clinical training/onboarding was typically done by physicians board certified in A&I or a combination of physicians and APPs experienced in A&I if already established at the practice/institution. Approximately 1/3 of respondents, particularly those at academic institutions, reported being trained in a niche of A&I; thus, they were seeing primarily patients with food allergies or asthma etc. APPs reported a range of comfort levels at completion of their onboarding experience that appeared proportional to the rigor/length of training received.

Five physicians who had recently onboarded an APP to their A&I practice/institution participated in qualitative interviews. As observed in the APP experience, clinical training was performed by physicians or a combination of physicians and APPs experienced in A&I if present at the practice/institution. The majority reported a strong need for an APP dedicated curriculum. Formal educational sessions typically consisted of modifying lectures initially prepared for physician fellows to a level more appropriate for APPs, while noting this was suboptimal. The time needed to clinically onboard an APP within A&I specifically was also considered significant and difficult to balance with ongoing clinical needs.

Quantitative Results: Fifty-six APPs within the AAAAI Allied Health Committee completed the targeted needs assessment out of 140 total APP members (40% completion rate). Results are summarized in Table 1 with full survey data available in the Dryad link available below. Respondents were typically well experienced in A&I with 44% in practice for 11 or more years and only 11% in practice for 3 years or less. Similarly, the majority were practicing in academic medical centers (56%) with 37% in private practices. Regarding training specific to A&I in NP/PA school, the majority of respondents reported receiving limited training in allergy (50%) and immunology (54%) with 68% of individuals reporting they either strongly disagreed or somewhat disagreed that training specific to A&I in NP/PA school prepared them for clinical practice specializing in A&I.

**Table 1.** Quantitative Results of Targeted Needs Assessment Survey.

Question	Response # (%)
Duration as an APP in A&I?	• <1 year 1 (2%) • 1–3 years 5 (9%) • 4–5 years 10 (18%) • 6–10 years 14 (26%) • 11+ years 24 (44%)
Practice Setting	• Private Practice 20 (37%) • Academic Medical Center 30 (56%) • Private Practice 1 (2%) Affiliated with an Academic Center • Other 3 (5%)
Which characterizes your training in Allergy in NP/PA school (definitions of responses listed below)	• None 9 (17%) • Limited 27 (50%) • Moderate 15 (28%) • Extensive 3 (6%)
Which characterizes your training in Immunology in NP/PA school (definitions of responses listed below)	• None 19 (35%) • Limited 29 (54%) • Moderate 5 (9%) • Extensive 1(2%)
Training specific to A&I in NP/PA school prepared me for clinical practice	• Strongly disagree 19 (35%) • Somewhat disagree 18 (33%) • Neither agree nor disagree 8 (15%) • Somewhat agree 8 (15%) • Strongly Agree 1 (2%)
Number of APPs at practice/institution within A&I	• 1 14 (26%) • 2–4 28 (52%) • 5–10 8 (15%) • 10 4 (7%)
Which describes the amount of formal education received at practice site when starting in A&I? (definitions of responses listed below)	• None 10 (19%) • Limited 12 (23%) • Moderate 8 (15%) • Extensive 23 (43%)
Which describes the duration of clinical training in A&I before practicing at current level of independence?	• 0–4 weeks 7 (13%) • 1–3 months 6 (11%) • 3–6 months 14 (25%) • 6–9 months 11 (21%) • 9–12 months 0 (0%) • 12–24 months 6 (11%) • 24+ months 9 (17%)
Which best characterizes your comfort level in seeing patients after completion of your training period?	• Extremely uncomfortable 2 (4%) • Somewhat uncomfortable 7 (13%) • Neither comfortable nor uncomfortable 1 (2%) • Somewhat comfortable 28 (53%) • Extremely comfortable 15 (28%)
Training/education in A&I at clinical practice site was sufficient in preparing you for practice	• Strongly disagree 0 (0%) • Somewhat disagree 5 (9%) • Neither agree nor disagree 2 (4%) • Somewhat agree 17 (32%) • Strongly agree 30 (55%)
Legend. Definitions of choices provided to participants: • Which characterizes your training in Allergy/Immunology in NP/PA school ◦ Limited: 1–2 lectures ◦ Moderate: Several lectures with moderate clinical exposure ◦ Extensive: robust curriculum with comprehensive clinical exposure • Which describes the amount of formal education received at practice site when starting in A&I ◦ Limited: 1–4 lectures ◦ Moderate: 5–10 lectures ◦ Extensive: 10+ lectures	

There was wide variation in the clinical training/onboarding experience of APPs to A&I at their initial clinical practice/institution. As noted in the gap analysis, APPs were typically the only APP practicing A&I at their institution (24%) or part of a small group of 2–4 APPs total (52%). There was significant variation in the amount of formal education received with 19% receiving no formal didactic education up to 43% receiving extensive education. Similarly, there was variation in the duration of clinical training before practicing at their current level of independence with 13% receiving 0–4 weeks, 11% 1–3 months, 25% 3-6 months, 21% 6–9 months, 11% 12–24 months and 17% 24+ months. Upon completion of onboarding, the majority (53%) reported they felt somewhat comfortable seeing patients at their specific level of independence. The majority somewhat agreed (32%) or strongly agreed (55%) that their onboarding to A&I sufficiently prepared them for clinical practice.

When asked qualitatively what was missing from their training experience in A&I, frequent responses included a formal curriculum specific to APPs in A&I, lack of resources geared towards APPs new to A&I, immunology training, mentorship/education from APPs with experience in A&I, ongoing mentorship/training from MD’s and APPs and a fellowship specific to A&I for APPs.

## Discussion

Results of a preliminary gap analysis and targeted needs assessment of APPs practicing in A&I identified that training in NP/PA school specific to A&I is minimal, consisting of 1–2 lectures reported by most respondents. However, this is to be expected as similar to medical school for physicians, comprehensive training in every subspeciality is not feasible. Similar to the residency requirement for physicians, literature has demonstrated that new APPs benefit from TTP programs to successfully transition them into their roles ^12– 15^ with TTP programs associated with greater practice autonomy, job satisfaction, clinical productivity, and decreased turnover ^16– 18^. TTP programs consist of residency/fellowships that last 1–2 years and onboarding programs which are shorter duration (usually 6–12 months) and do not provide a formal credential or certificate upon completion ^12^. Both programs typically include a gradual increase in clinical responsibilities accompanied by mentoring and educational sessions. To date, residency/fellowships for APPs have been limited to larger academic medical centers or more resourced practices ^12^. A recent scoping review of all TTP programs for APPs noted an increase in publications on such programs in recent years with most published programs being in the hospital/inpatient setting ^12^. Examples of established residency/fellowships for APPs include hematology/oncology, neonatology, surgical subspecialities and hospital medicine ^19– 21^. At present, there is no residency/fellowship for APPs within A&I and no publications describing a structured onboarding program ^6^.

Evaluation of the current onboarding/educational training of APPs new to A&I in our gap analysis and targeted needs assessment demonstrated a wide range in experiences at their initial practice site within A&I with 49% receiving ≤6 months of clinical training which is less than the typical 6–12 months described in TTP onboarding programs in other specialties. The range of formal education each APP received also varied, with 42 % reporting receiving none or limited formal education specific to their role during onboarding. Most APPs reported they only felt somewhat comfortable after completion of training, which likely represents a current gap in the onboarding process at select institutions/practices. The variation new APPs to A&I are receiving demonstrates that standardized of onboarding processes is essential to ensure adequate training to the field.

Frequently cited educational needs in both the gap analysis and in the open-ended responses in the targeted needs assessment included introductory level material for an APP new to A&I, a residency/fellowship for APPs, continuing education specific to APPs and additional immunology training. This provides insight into development of our intended Core Curriculum for APPs in A&I and also sets the stage for future educational efforts specific to APPs in A&I.

There are significant limitations of this study. Participants were recruited from the AAAAI Allied Health Committee which is unlikely to be representative of all APPs practicing in A&I as it likely overrepresents those in academic medicine. However, the gap analysis participants were intentionally balanced for site of practice and 35% of those who completed the targeted needs assessment were in private practice. Similarly, a large portion of the respondents were highly experienced in the field with 45% being in A&I for 11+ years which also likely significantly impacts responses. There is unfortunately no universal list serve or available accurate tally of the total number of APPs in A&I given the absence of a centralized licensing body like the American Board of Allergy & Immunology for physicians.

In conclusion, results of a gap analysis and targeted needs assessment of APPs in A&I identify a wide range in onboarding/formal clinical training in A&I, specifically at their initial practice site. The majority of APP’s only felt somewhat comfortable in their role after completion of training. Our findings support the need for further educational efforts for training APPs in A&I as has been done in other specialties such as a targeted curriculum specific to APPs within A&I, a standardized onboarding process and potentially a dedicated residency/fellowship for APPs in A&I.

## Ethics and consent

This educational project was determined to be exempted from IRB oversight by the University of Colorado School of Medicine. As for the IRB exemption, it was determined on 11/5/2024. Since they exempted it from research ethics review there is no IRB reference number but the internal number within the IRB for the request is #255054. Given the study was deemed exempt, written informed consent was not required, as this was an educational project in which sensitive information was not obtained.

## Data Availability

Dryad: Needs assessment of advanced practice provider education in allergy & immunology, https://doi.org/10.5061/dryad.fxpnvx127 ^9^ This project contains the following underlying data: Data for Publication. Updated README Data are available under the terms of the Creative Commons Zero "No rights reserved" data waive (CC0 1.0 Public domain dedication).
