# Supplementary material for: A Case for Specific Education of Advanced Practice Providers in Allergy & Immunology: Results of a Gap Analysis and Targeted Needs Assessment
Source: MedEdPublish (2016). 2025 Apr 3;14:236. Originally published 2024 Oct 17. [Version 2] doi: 10.12688/mep.20586.2 (PMC11976216; doi:10.12688/mep.20586.2)
Supplement: Peer review history

# Reviewer report: Reviewer response for version 2

**DOI:** 10.21956/mep.22374.r41335

**Published:** 2025-04-07

**Authors:** Jennifer Mammen

**Affiliations:** 1 University of Massachusetts Dartmouth, Dartmouth, Massachusetts, USA

I appreciate the substantial revisions made to this manuscript.  The current revision does a nice job of  addressing prior weaknesses and methodological concerns.  Methods are now clearly presented and reflected in the results.  The discussion appears balanced and collegial, while highlighting important gaps in APP education with useful suggestions on how these gaps could be addressed. 

 Thank you for the opportunity to review this manuscript for the second version.

Have any limitations of the research been acknowledged?

No

Is the study design appropriate and does the work have academic merit?

Partly

Is the work clearly and accurately presented and does it cite the current literature?

No

If applicable, is the statistical analysis and its interpretation appropriate?

No

Are all the source data underlying the results available to ensure full reproducibility?

No

Are the conclusions drawn adequately supported by the results?

No

Are sufficient details of methods and analysis provided to allow replication by others?

No

Reviewer Expertise:

Asthma, Symptom science, NP education, Parkinson's, Symptom mapping

I confirm that I have read this submission and believe that I have an appropriate level of expertise to confirm that it is of an acceptable scientific standard.

# Reviewer report: Reviewer response for version 1

**DOI:** 10.21956/mep.22033.r39915

**Published:** 2024-12-16

**Authors:** Polat Goktas

**Affiliations:** 1 University College Dublin, Belfield, Country Dublin, Ireland

Thank you for submitting your manuscript titled “ *A Case for Specific Education of Advanced Practice Providers in Allergy & Immunology: Results of a Gap Analysis and Targeted Needs Assessment*” for review. However, I have major comments that need to be addressed to enhance the clarity, rigor, and impact of your work.

**Introduction:**

- Spell out terms like A&I and APPs on first use for clarity.
- Explain systemic reasons for increased APP utilization, such as cost-effectiveness and physician shortages.
- Provide references or evidence for the lack of a nationally sanctioned curriculum in A&I.
- Include relevant studies on APP training to strengthen the background.
- Clarify the link between state regulations and the absence of a national curriculum; focus on standardization issues.
- Connect the introduction to the study’s objective, emphasizing the importance of addressing gaps in APP education.

**Methods:**

- Clearly describe the data collection tools.
- Recruitment via the AAAAI Allied Health Listserv introduces bias; acknowledge this limitation and its impact on generalizability.
- Methods describe qualitative interviews, but results are mostly quantitative; clarify the study design (qualitative, quantitative, or mixed).
- Provide specifics on inclusion criteria, participant numbers, and response rates.

**Results:**

- Results present quantitative data, but the methods describe qualitative approaches. Ensure consistency between methods and results.
- Percentages and trends are reported without context. Provide clear explanations of what the data implies.
- Mentioned open-ended responses lack themes or participant quotes. Summarize key insights and include supporting quotes.
- Claims of statistical significance lack p-values or confidence intervals. Add these for validation.
- Explain vague terms like “ *extensive training*” and provide more context.

**Discussion:**

- Avoid overgeneralizations like “ *significant training gap*” without clear evidence. Tie conclusions directly to results.
- Compare A&I training gaps to other specialties for context.
- Propose actionable steps for designing and implementing a dedicated curriculum.
- Integrate relevant studies to strengthen arguments and situate findings within the field.

Have any limitations of the research been acknowledged?

Partly

Is the study design appropriate and does the work have academic merit?

Partly

Is the work clearly and accurately presented and does it cite the current literature?

Partly

If applicable, is the statistical analysis and its interpretation appropriate?

Partly

Are all the source data underlying the results available to ensure full reproducibility?

Yes

Are the conclusions drawn adequately supported by the results?

Partly

Are sufficient details of methods and analysis provided to allow replication by others?

Partly

Reviewer Expertise:

My research focuses on allergy and immunology, with an emphasis on integrating digital technologies into education and practice.

I confirm that I have read this submission and believe that I have an appropriate level of expertise to confirm that it is of an acceptable scientific standard, however I have significant reservations, as outlined above.

# Reviewer report: Reviewer response for version 1

**DOI:** 10.21956/mep.22033.r40044

**Published:** 2024-11-29

**Authors:** Paneez Khoury

**Affiliations:** 1 National Institutes of Health, Bethesda, USA

There is a perceived gap in structured curriculum for APPs in A&I which may both impact APPs and physicians working with them, alike. Through a needs assessment and qualitative approach, the authors note that training varies widely and that regulations by state may impact requirements. The introduction suggests that there is a codified curriculum for physicians (there is not) in Allergy-Immunology. Instead, the governing structure for assessing competence in many specialties are built, in part, on items that are felt to be important for board passage. In general, knowledge-based content is defined on the needs/particularities of the program/institution and the patient population they serve. In addition, the authors do not mention differences between medical knowledge domains and the need for procedural readiness. Clearly, there is a desire for more structured learning based on the gap analysis and this may improve preparation and desire for NP/PAs to enter the field of A&I.

**There are several opportunities to address elements that would improve the thrust and conclusions from this report:**

1. **Is **there an estimate of the number of APPs practicing in A&I as this would inform the needs assessment and educational changes on the part of APP programs.
2. Given the inclusion of a select group of APPs participating in an academic society, what do they authors think might improve the generalizability? Is there a similar group within the ACAAI, another society where APPs may be members? What about perceptions of NP/PAs currently in school who contemplate a career in A&I?
3. The authors combine NP/PA in one category; however, in effect these roles are operationally different in terms of need for supervision and independent practice (depending on state). Both clinical training and hiring mechanisms are different also. It doesn’t appear from the data provided that those differences were captured? Was location/state level data collected.
4. Are authors able to provide a Table 1 showing respondent characteristics (beyond years in practice?) Age/sex/NP/PA (as in comment 3).
5. Its hard to compare perception of comfort level from a few years out of training to those practitioners who are 11 years out? In the qualitative interviews was comfort in independent practice, comfort in medical knowledge, or procedural skills collected?
6. I don’t fully understand the figure, the x axis in particular is unclear which is clinical training and which is time in field.  Please provide a more descriptive title and figure legend is provided to more clearly understand the stacked bars.
7. There is mention of “trends” but no statistics or table of results provided for figure 1.
8. The interpretation of the figure may also be somewhat flawed. Was “clinical training” defined as only during NP/PA school, or did informal clinical training count? If the latter then one could imagine that apprenticeship models could have played a larger role in NP/PA definition of ‘training’.
9. It appears that 40/140 respondents completed the initial survey. How were the respondents selected for the qualitative interviews?
10. There is mention of physician respondents: “Among physicians, most reported a strong need for an APP dedicated curriculum with physicians typically modifying lectures initially prepared for physician fellows to a level more appropriate for APPs, while noting this was suboptimal.” How was this survey administered and how were the physicians selected?
11. Please provide the original questionnaire, the interview guide for the qualitative interviews and the modified Delphi questions in an appendix (if allowable by the journal).
12. The results are laid out in two paragraphs and primarily outline percentages of comfort and types of lectures. This could more easily be presented in a table format and the results of the qualitative interviews and needs assessment better represented in the results.
13. If the results of the needs assessment are taken at face value, what are next steps in view of the authors? This could be better presented in the discussion. Authors state: “Therefore, a similar duration of training in A&I is likely needed.” Suggesting need for codified fellowship training; however, its unclear how this would come about or how they would be sponsored.

Have any limitations of the research been acknowledged?

Partly

Is the study design appropriate and does the work have academic merit?

Yes

Is the work clearly and accurately presented and does it cite the current literature?

Partly

If applicable, is the statistical analysis and its interpretation appropriate?

Not applicable

Are all the source data underlying the results available to ensure full reproducibility?

Partly

Are the conclusions drawn adequately supported by the results?

Partly

Are sufficient details of methods and analysis provided to allow replication by others?

No

Reviewer Expertise:

Allergy Immunology, Medical Education

I confirm that I have read this submission and believe that I have an appropriate level of expertise to confirm that it is of an acceptable scientific standard, however I have significant reservations, as outlined above.

# Reviewer report: Reviewer response for version 1

**DOI:** 10.21956/mep.22033.r39433

**Published:** 2024-10-30

**Authors:** Jennifer Mammen

**Affiliations:** 1 University of Massachusetts Dartmouth, Dartmouth, Massachusetts, USA

**MedEdgePublish Review**

 A Case for Specific Education of Advanced Practice Providers in Allergy & Immunology: Results of a Gap Analysis and Targeted Needs Assessment [version 1; peer review: awaiting peer review]

 This report describes the result of an assessment of NP/PA training in the areas of asthma, allergy, and immunology and readiness for practice.  I appreciate the effort that has gone into this work, and I agree that this is an important topic in need of attention.  I also agree that there is likely insufficient training in this area.  However, I regret to say that there are major scientific flaws in this study, and this should not be represented as a gap analysis.  Reframing this as preliminary work that suggests a lack of preparedness in asthma and immunology training for NP/PA might be the best way to go. This study does provide some useful information but based on the methodological approaches should be treated as preliminary work preparatory to a more rigorous national survey.  I would also suggest adjusting the tone of the manuscript to sound less devaluing of the NP/PA professions.  As written, this is likely to be offensive. 

**Is the work clearly and accurately presented and does it cite the current literature?**

- **Sentence**:  “NPs are increasingly utilized throughout the health care system” –may wish to include cost effectiveness as this is another important reason for increasing use of NP.
- First mention of A&I in text should be spelled out prior to using the acronym.
- Sentence does not make sense: “At present a nationally sanctioned A&I curriculum specific to APPs within A&I is lacking, likely due in part to state-by-state regulatory requirements that dictate the scope of APP practice.”  It would be more logical to state that it is due to standards and requirements of certifying bodies is as this is typically what drives curriculum.  A&I is well within scope of practice.

**Is the study design appropriate and does the work have academic merit?  Are sufficient details of methods and analysis provided to allow replication by others?**

- There is insufficient methodological description and what is included appears questionable.
- Major limitation in the sampling strategy:  Participants were recruited through the American Academy of Allergy, Asthma & Immunology (AAAAI) Allied Health List serve.  This means that only participants who were part of this network, would have been recruited, which biases the sample towards a very small group of participants with a preexisting interest in A&I.  Sampling base was 140 NP/PA in the AAAAI network, of which a small number responded.)  As such, findings in this report are unlikely to represent the preparedness of diverse NPs across the country.  This should be clearly addressed in the discussion.
- Major limitation in methods:  The methods section does not describe the study methodology in sufficient detail for replication.  The methods do not align with the results presented.  The methods start to describe a qualitative study, but mostly quantitative results are presented.  There is no description of the survey used, the interview guide or process, or data analysis procedures. **Approaches do not align with the results.

**If applicable, is the statistical analysis and its interpretation appropriate?**

- Not clearly described and cannot assess – missing both the statistical and QDA descriptions.
- The interpretations seem inappropriate without evidence to substantiate.

**Have any limitations of the research been acknowledged?**

- The limitations are not sufficiently addressed.

**Are all the source data underlying the results available to ensure full reproducibility?**

- This is not reproducible based on details provided.

**Are the conclusions drawn adequately supported by the results?**

- The conclusion are not supported by methodology or evidence.

**Other comments**

- Please clarify what is meant by “There was a wide range of education received at practice sites (1–2 weeks to 24+ months)”.
- **Sentence**: *“Among physicians, most reported a strong need for an APP dedicated curriculum with physicians typically modifying lectures initially prepared for physician fellows to a level more appropriate for APPs, while noting this was suboptimal.”*  I would suggest revising this and clarifying what you mean here. Physicians typically do not teach in nurse practitioner programs, and this statement would not be helpful to improving nurse practitioner programs.
- **Sentence**: *“The time needed to train an APP was also considered significant which was difficult to balance with ongoing clinical needs.”  *I have similar concerns regarding this sentence. I am not clear what the authors are talking about here. Are you talking about NP curriculum or post graduation education provided by physicians to nurse practitioners that they believe are suboptimally trained? Or is this specific to PA education?  This sounds highly critical and does not blend well with current educational practices.
- **Sentence**: *“Fifty-six APPs within the AAAAI Allied Health Committee completed the targeted needs assessment out of 140 total members”.*  This does not seem to align with the methods described. You indicated that you completed 20 interviews with APP's and 5 with physicians. Then in the results an entirely different approach is presented that was not described in the method section.
- **Sentence**:  " *Interestingly, there was a statistically significant trend towards APPs who more recently graduated NP/PA school having less clinical training before practicing at their current level of independence than those who have been in practice 6 or more years. While the reasons for this are unclear, financial and logistical factors such as patient wait times and physician bandwidth to train an APP may be contributing.” * I have similar strong objections to the conclusions regarding  “patient wait times and physician bandwidth to train APP” in the second sentence.  Does the authorship team include any NP/PAs?  If not, I strongly advise the authors to add both a doctorally prepared NP and PA as a co-author before resubmitting.  Whether intended or not, this paper is written in a way that appears disparaging and devaluing of the nurse practitioner and physician assistant professions.

Have any limitations of the research been acknowledged?

No

Is the study design appropriate and does the work have academic merit?

Partly

Is the work clearly and accurately presented and does it cite the current literature?

No

If applicable, is the statistical analysis and its interpretation appropriate?

No

Are all the source data underlying the results available to ensure full reproducibility?

No

Are the conclusions drawn adequately supported by the results?

No

Are sufficient details of methods and analysis provided to allow replication by others?

No

Reviewer Expertise:

Asthma, Symptom science, NP education, Parkinson's, Symptom mapping

I confirm that I have read this submission and believe that I have an appropriate level of expertise to state that I do not consider it to be of an acceptable scientific standard, for reasons outlined above.
